# When Red Turns Black: Influence of the 79 AD Volcanic Eruption and Burial Environment on the Blackening/Darkening of Pompeian Cinnabar

**DOI:** 10.1021/acs.analchem.1c02420

**Published:** 2021-11-24

**Authors:** Silvia Pérez-Diez, Africa Pitarch Martí, Anastasia Giakoumaki, Nagore Prieto-Taboada, Silvia Fdez-Ortiz de Vallejuelo, Alberta Martellone, Bruno De Nigris, Massimo Osanna, Juan Manuel Madariaga, Maite Maguregui

**Affiliations:** † Department of Analytical Chemistry, Faculty of Science and Technology, University of the Basque Country UPV/EHU, P.O. Box 644, 48080 Bilbao, Basque Country, Spain; ‡ Departament d’Arts i Conservació-Restauració, Facultat de Belles Arts, Universitat de Barcelona, Pau Gargallo, 4, 08028 Barcelona, Catalonia, Spain; § Institute of Electronic Structure and Laser − Foundation for Research and Technology, Nikolaou Plastira 100, Vassilika Vouton, 70013 Heraklion, Crete, Greece; ∥ Applied Research Laboratory of the Archaeological Park of Pompeii, via Plinio 4, 80045 Pompeii, Naples, Italy; ⊥ Former General Director of the Archaeological Park of Pompeii, via Plinio 4, 80045 Pompeii, Naples, Italy; # Director-General of the Directorate-General of Museums, via di San Michele 22, 00153 Rome, Italy; ∇ Unesco Chair on Cultural Landscape and Heritage, University of the Basque Country UPV/EHU, P.O. Box 450, 01008 Vitoria-Gasteiz, Basque Country, Spain; ○ Department of Analytical Chemistry, Faculty of Pharmacy, University of the Basque Country UPV/EHU, P.O. Box 450, 01008 Vitoria-Gasteiz, Basque Country, Spain; ¶ IAUB. Institut d’Arqueologia UB, Facultat de Geografia i Història, UB C/Montalegre 6-8, 08001 Barcelona, Catalonia, Spain

## Abstract

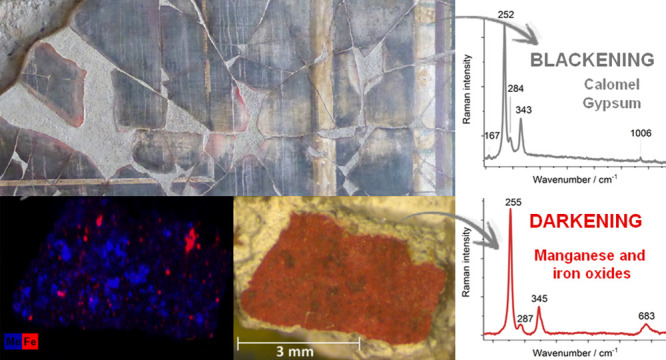

It is widely known that the vivid hue of red cinnabar can darken or turn black. Many authors have studied this transformation, but only a few in the context of the archeological site of Pompeii. In this work, the co-occurrence of different degradation patterns associated with Pompeian cinnabar-containing fresco paintings (alone or in combination with red/yellow ocher pigments) exposed to different types of environments (pre- and post-79 AD atmosphere) is reported. Results obtained from the in situ and laboratory multianalytical methodology revealed the existence of diverse transformation products in the Pompeian cinnabar, consistent with the impact of the environment. The effect of hydrogen sulfide and sulfur dioxide emitted during the 79 AD eruption on the cinnabar transformation was also evaluated by comparing the experimental evidence found on paintings exposed and not exposed to the post-79 AD atmosphere. Our results highlight that not all the darkened areas on the Pompeian cinnabar paintings are related to the transformation of the pigment itself, as clear evidence of darkening associated with the presence of manganese and iron oxide formation (rock varnish) on fragments buried before the 79 AD eruption has also been found.

The Roman city of Pompeii was destroyed after the eruption of Mount Vesuvius in 79 AD. Although this was an unfortunate natural and societal event, it resulted in a remarkably good conservation of its remains, thanks to the burial of the city under the pyroclastic flow. However, some of the pigments applied on the walls of Pompeii experienced transformations due to the eruption, such as the blackening process of hematite (α-Fe_2_O_3_)^1^ and the dehydration of yellow ocher (goethite, α-FeOOH) into hematite.^2,3^ A recent study has shown that another reason for the degradation of the mural paintings of Pompeii is the crystallization of salts coming from the pyroclastic materials ejected in the 79 AD eruption.^4^ In addition, since the first archeological excavations in the 18th century, the archeological park has suffered a continuous decay, due to its exposure to the modern atmosphere and the (former) application of restoration products that are no longer used.^5^

The study of ancient sources^6,7^ and archeological records demonstrates that red cinnabar (α-HgS) has been used as a pigment since antiquity. This precious pigment, employed in the mural paintings of the archeological site of Pompeii, suffers from blackening. Hence, Vitruvius did not encourage its application in open spaces (e.g., *peristylia*), since its exposure to sunlight and moonlight^6^ was already thought at that time to be responsible for its deterioration.

Prominent examples of cinnabar blackening are found at the Casa della Farnesina (Rome) or the Villa dei Misteri (Pompeii).^8,9^ This process occurs to a lesser degree in several locations and can remain unnoticed by nonexperts.

After visual inspection, the color of the altered cinnabar from Pompeii looks blacker^10^ than the one on other discolored cinnabar easel paintings.^11^ In the latter, the altered cinnabar/vermilion shows brownish to grayish hues.^11,12^

The blackening of cinnabar has traditionally been attributed to light exposure and transformation of red α-HgS (trigonal crystal system) into black β-HgS metacinnabar (cubic crystal structure), which is reported to take place at 344 ± 2 °C.^13^ However, there exist scarce confirmations of black metacinnabar detection in darkened cinnabar.^14,15^ Since Raman spectroscopy cannot distinguish cinnabar from metacinnabar, other techniques such as X-ray diffraction (XRD)^14^ or pump-probe microscopy^15^ could be applied for that purpose. Nevertheless, although different cinnabar-based mural paintings are exposed to light, not all of them show the same degree of transformation and even some areas do not present any sign of darkening or blackening.^8,11^ Hence, other variables, which could contribute to this transformation, should be considered for its complete explanation.

Further examples of the darkening or blackening process of cinnabar in presence of Cl in easel and mural paintings featuring mercury chlorides or Hg-S-Cl compounds (calomel: Hg_2_Cl_2_, mercury (II) chloride: HgCl_2_, corderoite: α-Hg_3_S_2_Cl_2_, terlinguaite: Hg_2_OCl, kenhsuite: γ-Hg_3_S_2_Cl_2_) have been published in the past years.^10,11,16−18^ Another plausible degradation pathway that involves the formation of gypsum crusts (possibly favored by the photodecomposition of cinnabar)^10^ has been proposed for the Vesuvian mural paintings of Torre del Greco (Campania), in which calcite acts as a binder: the formation of gypsum crusts as a result of calcite sulfation,^10^ favored by the photodecomposition of cinnabar. The subsequent accumulation of airborne particulate matter and organic pollutants inside the porous structure of gypsum gives the crust its black color. Cotte et al.^10^ mentioned that calcite sulfation could take place due to the influence of the SO_2_ present in the polluted atmosphere or to the oxidized S produced by the decomposition of HgS. This last hypothesis might explain the failure to identify black crusts (gypsum crusts with airborne particulate matter/organic pollutants) in murals from the Vesuvian area decorated with pigments, other than cinnabar.

In this work, blackened/darkened cinnabar paintings (alone or in combination with red/yellow ocher pigments) have been analyzed in situ and in the laboratory through a multianalytical methodology. The main goals of this study were (i) to determine the role of the 79 AD volcanic eruption in the blackening of Pompeian murals decorated with cinnabar and (ii) to evaluate whether different transformation phenomena can be identified on samples protected from the pre- and post-79 AD volcanic eruption.

To achieve these goals, three different kinds of cinnabar paintings were compared: (i) painted areas impacted by the 79 AD eruption, excavated more than 150 years ago and exposed to the modern atmosphere since then; (ii) painted panels impacted by the 79 AD eruption, removed during the excavations of the 19th century, stored at the Naples National Archaeological Museum (MANN) and thus, protected from the modern atmosphere; and (iii) painting fragments exposed to the ancient atmosphere of Pompeii, presumably detached after the 62 AD earthquake and deposited in a house pit since then.

## Experimental Section

### Samples and Studied Mural Paintings

Three Pompeian houses were selected for this study: House of Marcus Lucretius (Regio IX, 5, 3/24), House of Ariadne (Regio VII, 4, 31/51), and House of the Golden Cupids (Regio VI, 16, 7) (see Table S1). All the houses have suffered the influence of the volcanic eruption and the preserved mural paintings have been exposed to the modern atmosphere since their excavation (19th century to beginning of 20th century).

Two samples (ATT2007/14 and 16/56) from the *triclinium* of the House of Marcus Lucretius (see Table S1) were considered. In the wall paintings of this room, the blackening of hematite pigment was previously studied, being possible to identify the presence of coquimbite/paracoquimbite (Fe_2_(SO_4_)_3_·9H_2_O) as degradation product of the pigment.^1^ Interestingly, this house also presented a deposit where earlier detached mural decorations were abandoned and buried. This deposit was used to cast aside detached fragments, possibly as a consequence of the 62 AD earthquake that damaged the murals of the house.^19^ This waste pit was excavated during the EPUH (Expeditio Pompeiana Universitatis Helsingiensis) campaign in 2005. Since then, the recovered fragments have been stored in the dark. In this work, two fragments from this deposit (samples 3T, Red A) showing dark stains on the cinnabar painting layer were considered. Additionally, panel paintings extracted from the *triclinia* (panel references 9206, 9285, 8992, and 9103, the latter from the summer *triclinium*) in the excavations of the 19th century and stored since then at the MANN were also in situ analyzed. The three panels belonging to the *triclinium* are surrounded by a blackened red frame, which could have been painted with red cinnabar.

From the House of Ariadne, three samples were considered (samples 6, 17, and 18; see Table S1).

Finally, in the House of the Golden Cupids, the blackened cinnabar decorations from the *exedra* (Room G; see Table S1) were studied. Due to sampling restrictions in this house, the analyses were performed in situ, without taking any sample.

### Portable and Benchtop Instrumentation

The in situ molecular analysis was performed using a portable innoRam Raman spectrometer (B&W Tek, Newark, USA) equipped with a CleanLaze technology 785 nm excitation laser (<300 mW laser output power) and mounting the probe on a motorized tripod (MICROBEAM S.A. Barcelona, Spain). For the in situ elemental analysis, the XMET5100 (Oxford Instruments, UK) Handheld Energy Dispersive X-ray Fluorescence spectrometer (HH-EDXRF), equipped with an Rh X-ray tube, was used. Details about the normalization procedure to compare the S and Cl counts extracted from the walls and panels under study can be reviewed in the Supporting Information.

In the laboratory, the molecular study of the samples was achieved using the inVia confocal Raman microscope (Renishaw, Gloucestershire, UK). The main objective lens used was the 50× one. Excitation lasers of 785 (nominal laser power 350 mW) and 532 nm (nominal laser power 50 mW) were employed for the acquisition of the spectra. The spectra were acquired in the 60–1200 cm^–1^ or 60–3000 cm^–1^ spectral range and accumulated 3, 5 to 10 times for 5–10 s.

To confirm molecular results, an elemental imaging study was conducted on sample Red A. For that the M4 TORNADO (Bruker Nano GmbH, Berlin, Germany) EDXRF spectrometer was used. Elemental distribution maps were acquired at down to 25 μm of lateral resolution using a use of polycapillary lens and with the Rh X-ray tube working at 50 kV and 600 μA. The spectral acquisition and data treatment were performed using the ESPRIT software from Bruker.

To evaluate the composition of the black stains of the sample 3T, X-ray Photoelectron Spectroscopy (XPS) and Time-of-Flight Secondary Ion Mass Spectrometry (TOF-SIMS) were employed.

XPS analysis was conducted using a Thermo Scientific K-Alpha ESCA instrument equipped with aluminum Kα_1,2_ monochromatic radiation at 1486.6 eV. Neutralization of the surface charge was achieved by using both a low energy flood gun (electrons in the range 0–14 eV) and a low energy Ar-ions gun. Photoelectrons were collected from a take-off angle of 90° relative to the sample surface. The measurement was done in a Constant Analyzer Energy mode (CAE) with a 100-eV pass energy for survey spectra and 20-eV pass energy for high resolution spectra. Charge referencing was done by setting the lower binding energy C 1s photopeak at 285.0 eV C 1s hydrocarbon peak. Surface elemental composition was determined using the standard Scofield photoemission cross sections.

A TOF-SIMS IV instrument from Ion-Tof GmbH Germany was employed to collect the mass spectra and to conduct mapping. A pulsed Bi_3_ ion beam at 25 keV impacted the sample, the generated secondary ions were extracted with a 10 kV voltage, and their TOF from the sample to the detector was measured in a reflectron mass spectrometer. Pulsed Bi_3_ beam at 25 keV and incidence of 45° were used to scan 500 × 500 μm^2^ areas.

Additional details of the experimental aspects and data treatment conducted using specific benchtop and portable instruments are available in the Supporting Information.

## Results and Discussion

### Characterization of Blackened Cinnabar on Mural Paintings Impacted by the 79 AD Eruption and Nowadays Exposed to the Atmosphere

The eastern and southern walls of the *triclinium* of the House of Marcus Lucretius revealed the occurrence of Fe, Hg, and S, confirming the presence of cinnabar, together with red and yellow ochers.^20^ In situ Raman measurements allowed the systematic identification of calomel (Hg_2_Cl_2_) and gypsum (CaSO_4_·2H_2_O) on the blackened cinnabar areas. Cl was also identified by HH-EDXRF. Additional analytical details can be found in Table S2.

**Figure 1:**
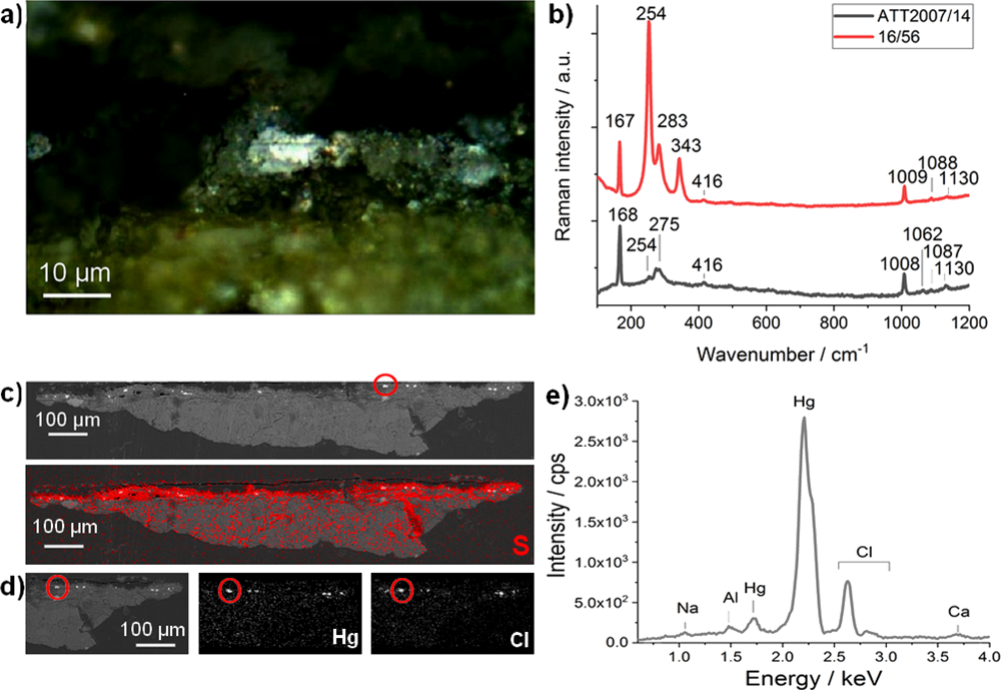
**(**a) Optical micrograph of sample 16/56 (particle circled in red in panels (c) and (d)). (b) Raman spectra of samples ATT2007/14 and 16/56 (north and south walls of the *triclinium*, House of Marcus Lucretius) showing the Raman bands of calomel (167/168, 275 cm^–1^), cinnabar (254, 283, 343 cm^–1^), gypsum (416, 1008, 1130 cm^–1^), wax (1062 cm^–1^), and calcite (1087 cm^–1^). (c) From top to bottom: BSE micrograph, and EDS map of 16/56 cross section showing the distribution of S. (d) From left to right: BSE micrographs of a detail of 16/56 cross section and distribution maps of Hg and Cl. (e) EDS spectrum acquired on the bright particle circled in red in panels (c) and (d).

To evaluate the presence of additional compounds in the *triclinium* of the House of Marcus Lucretius, samples from the northern wall (ATT2007/14) and southern wall (16/56) were analyzed in the laboratory by Raman microscopy (Table S2). Calomel was present in gray-whitish particles of sample 16/56 (see Figure 1a). In sample ATT2007/14, extracted from the upper red frame of the central panel, wax (band at 1062 cm^–1^), cinnabar (weak band at 254 cm^–1^), calomel (Hg_2_Cl_2_, bands at 168 and 275 cm^–1^), and gypsum (CaSO_4_·2H_2_O, bands at 1008 and 1130 cm^–1^) were detected (see Figure 1b).

The 1062 cm^–1^ Raman band was attributed to a wax applied in the 19th century restorations^5^ of the mural paintings and not to the presence of nitratine (NaNO_3_), based on the detection of a series of signals ascribable to an organic compound^21^ (see Figure S1, Supporting Information). The presence of the 1734 cm^–1^ band, assigned to the ν(C=O) vibrational mode, suggests the occurrence of a saturated wax. The proposed assignment of the rest of the Raman bands is shown in Table S3.

Both samples extracted from the *triclinium* of the House of Marcus Lucretius show a dark crust on the top of the painting layer (see for example the microscopic observation of sample 16/56, Figure 1a). To obtain further insights into the gypsum and calomel distribution on these samples, cross sections were studied by SEM-EDS. Figure 1a shows part of the stratigraphy of sample 16/56, composed of a “black crust” layer, a pictorial layer, and a plaster. An EDS map of the whole stratigraphy (Figure 1c) reveals the accumulation of S, attributed to the presence of gypsum in the “black crust” over the pictorial layer. In the latter, it was possible to detect bright particles (marked with a circle in Figure 1c,d) distributed throughout the layer. The EDS analyses (Figure 1e) confirmed the detection of both Hg and Cl in those particles (see Figure 1d), related to the presence of calomel. The cross section of the sample ATT2007/14 also revealed a black crust formed on the top of a pictorial layer with Hg-rich particles and chlorine.

**Figure 2:**
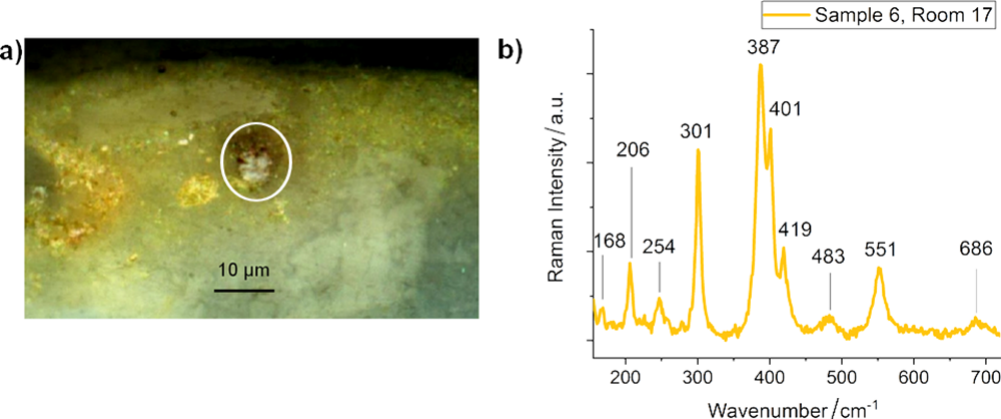
**(**a) Optical micrograph of sample 6 (southern wall of the *oecus*, House of Ariadne). (b) Selected Raman spectrum showing the bands of calomel, tridimite and goethite, and cinnabar acquired on the area circled in white.

In a preliminary in situ Raman screening from the area where sample 6 was obtained in the House of Ariadne, gypsum and calomel had been detected. This last was confirmed later in the laboratory with additional Raman analyses conducted on sample 6. Furthermore, microscopic observations allowed the identification of cinnabar as random pigment particles in a yellow pictorial layer, as in the case of the samples from the House of Marcus Lucretius (see Figure 2a). Raman analyses performed on these particles (see Figure 2b) showed the presence of goethite (FeOOH, bands at 301, 387, 483, 551, and 686 cm^–1^), related to the yellow color (yellow ocher), together with the presence of tridimite (high-temperature polymorph of SiO_2_, 206, 401, 419 cm^–1^), cinnabar (254 cm^–1^), and calomel (168 cm^–1^).

**Figure 3:**
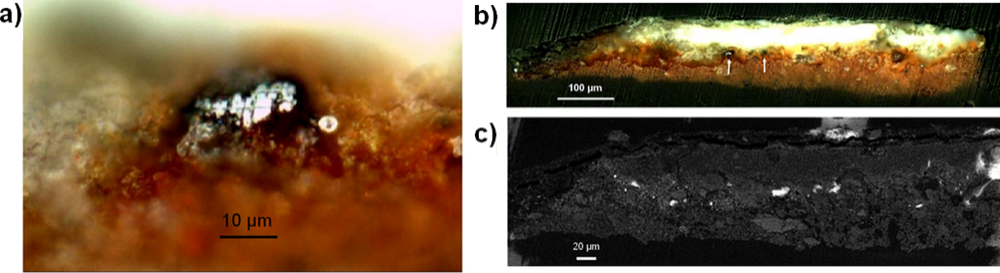
(a) Optical micrograph of the black-grayish metallic particles in sample 18 (*oecus*, House of Ariadne). (b) Optical micrograph of the cross section of the sample. (c) BSE micrograph of the cross section.

Calomel was also detected in sample 17, but not in sample 18. Gypsum had already been identified in situ by Raman spectroscopy. Sample 18 corresponds to a white stripe painted on a red background, which consisted of a mixture of cinnabar and red ocher (see Figure 3a,b). In this underlying pictorial layer, black-grayish metallic particles (around 15–20 × 5–10 μm^2^) were identified microscopically (Figure 3a,c). Raman and EDS spectra acquired on those particles did not offer additional information other than the signals related to cinnabar (Hg and S detection). Interestingly, a previous electrochemical study has demonstrated the formation of metallic mercury as a degradation product of HgS upon the influence of light and Cl^–^,^22^ whereas a recent publication concerning egg tempera painting has already proposed the occurrence of metallic mercury on vermilion mock-ups.^23^

The attributions of the in situ and laboratory-based Raman spectra of the House of Ariadne are summarized in Table S2.

**Figure 4:**
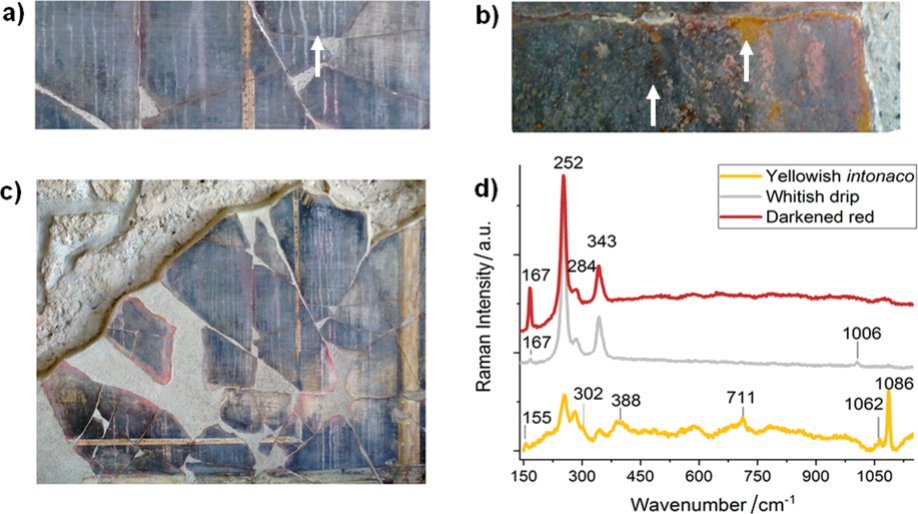
In the House of the Golden Cupids: (a) Detail of the whitish drips caused by rainwater percolation. (b) Close-up view of the underlying yellow plaster. (c) General view of the blackened *predella* of the southern wall. (d) Selected Raman spectra of the blackened cinnabar of the *exedra*, the whitish drips, and the underlying yellow plaster, marked with arrows in Figure 4a,b. The spectra show the Raman bands of calomel (167 cm^–1^) and cinnabar (252, 284, 343 cm^–1^); calomel, cinnabar, and gypsum (1006 cm^–1^); and cinnabar, calcite (155, 711, 1086 cm^–1^), goethite (302, 388 cm^–1^), and a protective wax (1062 cm^–1^).

The cinnabar used in the southern wall of the *exedra* of the House of the Golden Cupids shows a totally black appearance, suggesting that the blackening process is even more dramatic than the one occurring in the well-known Villa dei Misteri^9^ (see Figure 4a-c). In the painting fragments of the *predella*, the *intonaco* described by Meyer-Graft,^24^ based on a yellowish granular lime with fine orange inclusions, is visible in some areas (see Figure 4b). The Raman analysis of this mortar lead to the identification of calcite (155, 711, 1086 cm^–1^) and goethite (302, 308 cm^–1^). In addition, the 1062 cm^–1^ Raman band could correspond to a wax, as in the case of the House of Marcus Lucretius (see Figure S1 and Table S3), probably applied to the painting during the 20th century restorations of the house.^24^

The in situ measurements performed on the totally blackened cinnabar from the *predella* showed the ubiquitous presence of calomel and gypsum, together with cinnabar (see Figure 4d). Both calomel and gypsum were also detected in the whitish drips (see Figure 4a). In a previous study, thanks to portable laser-induced breakdown spectroscopy (LIBS) mapping of the mural paintings of the House of the Golden Cupids,^25^ it was possible to assess that this *predella* was the most Cl-impacted painted surface among those considered in the study.

### Characterization of Blackened Cinnabar on Panel Paintings Stored at Naples National Archaeological Museum (MANN)

Three panel paintings (9206, 9208, and 9255) extracted from the *triclinium* of the House of Marcus Lucretius include a red frame that nowadays looks quite blackened (see Figure S2). HH-EDXRF measurements conducted in all the frames allowed the detection of Hg and S together with high Fe contribution. These results pointed out to the combined use of red ocher and cinnabar. Bands associated to calomel or other Hg-Cl or Hg-S-Cl compounds were not identified in any of the in situ Raman measurements performed on the blackened frames. However, Cl was detected by HH-EDXRF in the blackened frames (see the example of panel 9206 in Figure S2).

Gypsum was also detected in the blackened cinnabar areas (see the example of panel 9103 in Figure S3) of all the considered panel paintings.

Interestingly, the identification of gypsum was not only restricted to the blackened cinnabar areas. This sulfate has been previously identified by infrared spectroscopy on the same panel paintings.^26^

To discard the intentional addition of gypsum to the plaster, several measurements were conducted on the surface of the panel paintings 9285, 9206, and 8992 by HH-EDXRF. Moreover, additional measurements were performed on the south and east walls of the *triclinium*, concretely on the surrounding areas (left and right side) of the voids that the panels left when they were removed during the first excavations of the house (see Table S4 and Figure S4). The normalized net counts of S and Cl obtained from each spectrum of each panel paintings stored in the MANN were compared with the ones obtained from the measurements in the walls from Pompeii. Since the red frames are rich in HgS, these points were not taken into account for the evaluation of S originating from gypsum. According to the obtained values, the normalized counts of S are higher in the panel paintings preserved at MANN, than in their respective adjacent walls currently exposed to the modern atmosphere (see Table S4 for comparison).

The S decrease in the exposed walls may be associated to the dissolution-mobilization-recrystallization of the formed sulfates during the exposure to the open atmosphere. Moreover, the restoration campaigns conducted in this room could have also contributed to the reduction of the content of soluble salts such as sulfates in the wall.

The S intensities are lower in the walls of Pompeii nowadays exposed to the atmosphere than in the panel paintings preserved since the first excavations around 170 years ago. Thus, it can be affirmed that, in the *triclinium* of the House of Marcus Lucretius, the current atmospheric SO_2_ is not playing a crucial role in the sulfation of the calcium carbonate of the wall paintings.

As regards the normalized Cl counts (see Table S4), they are only slightly lower in the panel paintings (0.3 ± 0.1 in panel 9206) than in the exposed walls (0.9 ± 0.3 in the left side of panel 9206, 0.5 ± 0.2 in the right side of panel 9206). The only exception is the right side of the void left by panel 9191 on the southern wall (3.7 ± 0.3). In this area, a prolonged direct exposure to the marine aerosol is expected (see Figure S5, Supporting Information) due to the strikingly intense Cl peak. On the other hand, low Cl intensities, present both in the stored panels and in the exposed walls, may be attributed to the Cl emission of the volcanic eruption^27^ and/or to a diffuse exposure to marine aerosol.

Note that in some cases the standard deviation related to Cl and S normalized counts is high (see Table S4). This is associated with their heterogeneous distribution in the walls.^25^

### Characterization of Dark Stains on Cinnabar in Painting Fragments Buried and Protected from the 79 AD Eruption

Samples 3T and Red A, recovered from the deposit of the House of Marcus Lucretius, were also studied to observe possible differences in the state of conservation of cinnabar not exposed to the 79 AD eruption and to the atmosphere since their recovery from the excavations.

In this case, the samples did not show a widespread black appearance of the cinnabar layer, but only certain dark stains or patches (see the species identified by Raman spectroscopy in this work in Table S2). In previous studies conducted using Raman spectroscopy, calomel had been detected.^20^

Interestingly, the stratigraphic analysis of sample 3T shows that cinnabar was applied over a pictorial layer composed by Egyptian Blue (Raman bands at 112, 137, 164, 192, 378, 400, 431, 473, 568, 763, 788, 985, 1010, and 1083 cm^–1^)^28^ (see Figure S6) and goethite (300 and 386 cm^–1^, spectrum not shown). This suggests either a previous redecoration of the area from which these fragments were detached or the application of cinnabar as overlying color on a greenish blue background.^19^

Raman measurements performed on the dark spots (see Figure S7a, Supporting Information) unveiled the presence of a broad band at around 683 cm^–1^ (see an example of it in the measurements performed in sample Red A, Figure S7b). Bearing in mind that only cinnabar was clearly detected in this sample and no abundant evidences of hematite were identified, this band cannot be associated only with magnetite (Fe_3_O_4_),^29^ as it could happen in some measurements acquired in the darkened hematite areas of the *triclinium* in the House of Marcus Lucretius (Fe_3_O_4_, Raman band at 661 cm^–1^, see Figure S7c,d). Moreover, considering the width of the 683 cm^–1^ band, it is complicated to attribute it to a specific mineral phase being most probable the presence of a mixture of several ones.

To further investigate this issue, XPS and TOF-SIMS measurements were performed on sample 3T for an in-depth study of the dark patches (around 50–250 μm, see Figures S8 and 5). XPS was preferred for line analysis on altered (dark areas) and intact cinnabar areas, while TOF-SIMS was more adequate to perform mapping, due to the sample roughness and the better depth and lateral resolution of the technique.

**Figure 5:**
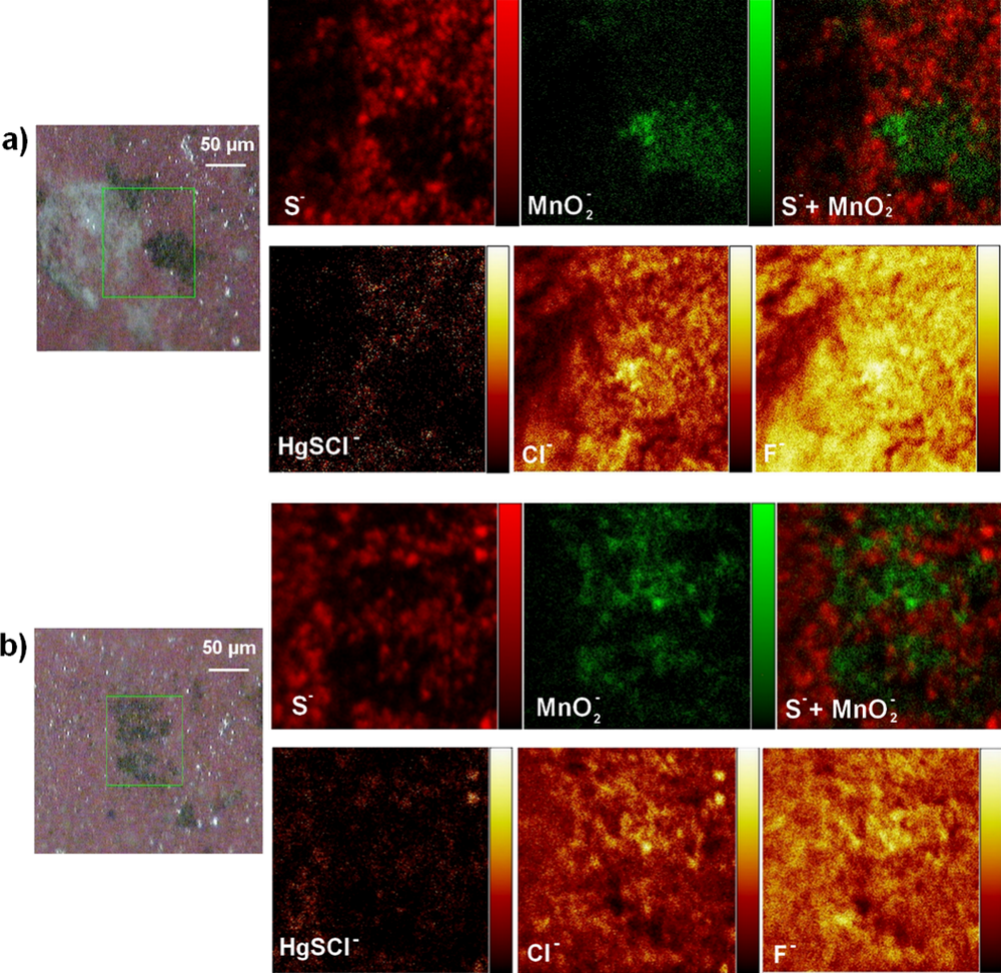
TOF-SIMS maps of negative ions (S^–^, MnO_2_^–^, HgSCl^–^, Cl^–^, F^–^) acquired in position 1 (a) and 2 (b) on sample 3T. In all scales, black color represents the absence of and specific lighter color represents a higher abundance of the negative ion represented.

The XPS analyses (see Figure S8) and TOF-SIMS maps (see Figure 5) on the dark stains revealed an increment in manganese and iron oxides or oxide hydroxides. Therefore, the broad band identified in the Raman measurements of the dark stains (see Figure S7, Supporting Information) could be related to the presence of a mixture of manganese and iron oxides or oxide hydroxides.^29−31^

In the literature, many references can be found regarding the formation of dark colored coatings composed mostly of manganese and iron oxides. This dark discoloration process is usually called “rock varnish”. Mn and Fe present in “rock varnish” could come from various sources, including dust and soil.^32,33^ Considering that these painting fragments have been buried for more than 2000 years, the occurrence of these metals could be readily elucidated. To explain the dissolution of Mn and Fe from the soil and their subsequent precipitation as oxides, pH and Eh changes in the burial environment should take place.^34^ Moreover, water should be present to favor the process. This is also guaranteed due to the previously assessed influence of groundwater in this archeological site.^4,25^

Whereas certain authors concluded that this phenomenon takes place under abiotic conditions, others held that microorganisms control Mn precipitation^35^ (biomineralization of Mn).

Although most of the “rock varnish” examples are located in desert environments,^32^ in the last years, different examples have been published regarding archeological contexts^36^ and 19th century buildings.^37^

Together with manganese oxides, whitish stains were also visible, related to the formation of a calcareous (calcite) patina (caused by the dissolution and recrystallization of the binder, see Figure S9). This result reinforces the influence of a water source in the dissolution–recrystallization process.

TOF-SIMS maps (area of 342 × 342 μm^2^) also showed the occurrence of F^–^ and Cl^–^ in the surface, while Hg-SCl^–^ (related to a Hg-S-Cl compound, such as corderoite, α-Hg_3_S_2_Cl_2_, or kehnsuite, γ-Hg_3_S_2_Cl_2_) was more abundant in the red areas not affected by the dark stains (see Figure 5). This result suggests that the presence of Cl^–^ is not always strictly related to the darkening of the cinnabar pigment, as already proposed by certain authors.^18^

The TOF-SIMS detection of F^–^, a halide of volcanic origin,^4^ reinforces the hypothesis of a leaching process (favored by groundwater) of the volcanic soil that covered the fragments, contributing to the increase in fluorine. Moreover, the contribution of groundwater rich in F^–^ and Cl^–^^4,25^ could also favor the relatively prominent presence of these halides in the cinnabar pictorial layer.

**Figure 6:**
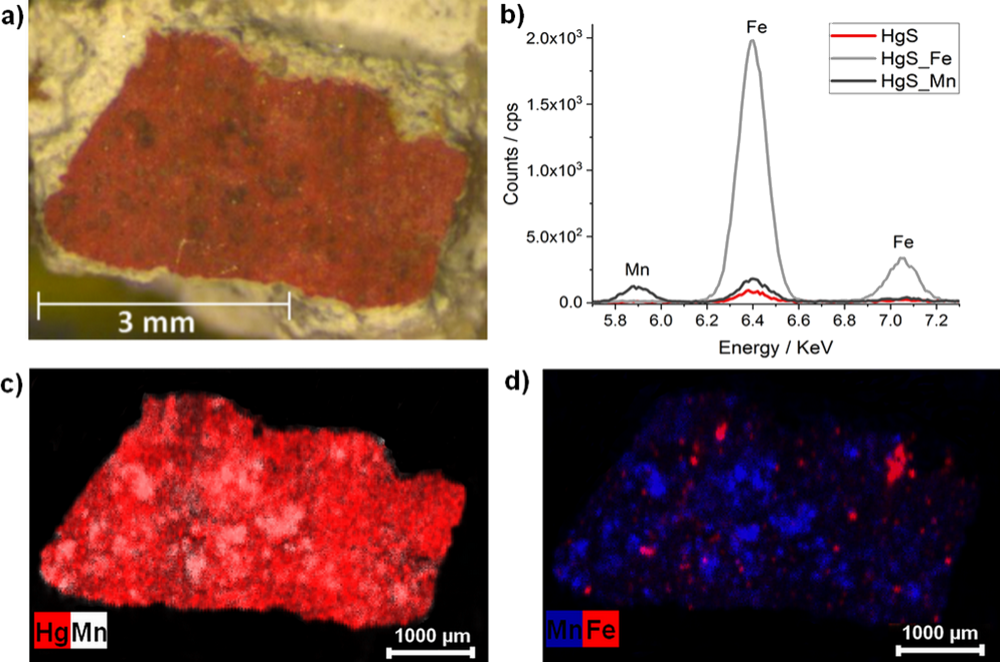
(a) View of sample Red A, featuring dark spots on the surface. (b) Selected μ-EDXRF spectra corresponding to the unaltered pictorial layer (HgS), a dark coating area rich in Fe (HgS_Fe), and a dark coating area rich in Mn (HgS_Mn). (c) μ-EDXRF elemental map showing the distribution of Hg and Mn on the fragment. (d) μ-EDXRF elemental map showing the distribution of Mn and Fe on the fragment.

To confirm the occurrence of manganese oxides on the dark patches of sample Red A detected by Raman spectroscopy, EDXRF imaging was conducted. As in sample 3T, the Mn distribution coincided with the dark areas present on the red cinnabar pictorial layer (Figure 6), verifying the presence of manganese oxides. Besides, Fe accumulations on these areas were detected, as expected according to the XPS analyses of sample 3T (Figure 6). In this case, Fe is scarcely distributed comparing with Mn, suggesting that iron oxide has been formed to a lesser extent over the cinnabar layer.

## Conclusions

This work shows how the state of conservation of the Pompeian cinnabar pigment varies depending on its protection against the pre- and post-79 AD atmospheres.

Gypsum has been systematically identified in the blackened areas of the pigment exposed to the pre- and post-79 AD atmospheres (e.g., House of the Golden Cupids). This result could suggest that the blackening might be related to the formation of a gypsum layer, either due to the polluted atmosphere or due to the sulfur emissions of the volcanic eruption. This layer would be subsequently enriched with airborne particulate matter, responsible for the final black color of the painting surface (“black crust” formation).

The experimental evidence agrees with what Cotte et al.^10^ previously suggested. In this last case, the sulfation of calcite in the fresco painting was explained by the oxidation of S coming from the decomposition of the HgS pigment. Nevertheless, in the mural paintings of the House of Marcus Lucretius here presented, the cinnabar proportion is much lower than the one of yellow ocher, and thus the extended formation of gypsum cannot be explained according to this hypothesis. In the future, additional painting stratigraphies, other than pure cinnabar or cinnabar mixed with ocher, should be investigated in order to track the formation of “black crusts” on other decorated/nondecorated areas of Pompeii.

The lower S intensities detected in the mural paintings of the *triclinium* of the House of Marcus Lucretius (exposed to the atmosphere since the 19th century excavations) compared with the panels of the same room preserved at the MANN, suggest that the H_2_S and SO_2_ emitted in the 79 AD eruption are crucial in the sulfation process.^26^ Another evidence of the impact owing to the eruption is the clear transformation of yellow ocher into red hematite in specific areas of the cubiculum annexed to the *triclinium* of this house.^2^ This room of the house is covered by a roof, protecting the mural paintings from the direct influence of polluted atmosphere, reducing the effects of this environmental agent in the sulfation process.

Regarding the protection of the cinnabar pigment when mixed with other pigments,^11,38^ this work demonstrates that cinnabar can be altered (calomel identification) even when blended with an ocher pigment. In addition, visually altered cinnabar particles were also identified in a red hematite pigment layer covered by a superficial one (calcite and dolomite). The absence of cinnabar transformation products in the metallic-like cinnabar particles identified could suggest that they either belong to metallic mercury, metacinnabar or even an amorphous cinnabar phase. This hypothesis should be confirmed in the future with the use of adequate instrumentation, which can offer sub-micrometric resolution, such as synchrotron assisted μXANES at the Hg L3-edge.

In the buried Pompeian cinnabar-based fresco fragments and not exposed to the 79 AD eruption, well preserved areas and dark stains/patches were identified. In the nondarkened areas Hg-Cl and Hg-Cl-S compounds were detected. These results confirm that such compounds can be formed independently of the pigment darkening or blackening process, as already stated by various authors.^18^ Moreover, in the darkened and not darkened areas of the samples, it was not possible to identify the presence of gypsum, since they were exposed neither to the H_2_S and SO_2_ gases of the eruption nor to the postexcavation atmosphere. On the contrary, the dark patches/stains are rich in manganese and iron oxide hydroxides, and do not belong to the conventional blackening process of the cinnabar. Therefore, for conservation purposes, when a cinnabar mural painting/fragment is recovered from an archeological context, an in-depth characterization of the dark/black formations on the cinnabar is necessary to conclude whether the cinnabar pigment is transformed or just affected by “rock varnish” or by the precipitation of other colored crusts.

Furthermore, this work also demonstrates that the color of the transformed Pompeian cinnabar may suggest different pigment degradation prompted by the impact of a number of environmental agents. The main transformation occurred after its exposure to the pre- and post-79 AD atmosphere is the blackening process connected to the formation of calomel and gypsum. On the other hand, buried Pompeian cinnabar could experience darkening due to the formation of black/brownish Mn/Fe stains and not to the raw pigment transformation itself.

In the future, accelerated weathering experiments using cinnabar fresco mock-ups reproducing the pre/post-79 AD atmosphere impact and burial environment will help delve into the chemical reactivity leading to these transformation products. It will thus allow the development of conservation protocols, which will protect and preserve the original red color of this pigment.

## Supporting Information Available

Additional experimental details, description of the samples and paintings analyzed and the obtained results (PDF)
