# Supplementary material for: When Red Turns Black: Influence of the 79 AD Volcanic Eruption and Burial Environment on the Blackening/Darkening of Pompeian Cinnabar
Source: Anal Chem. 2021 Nov 24;93(48):15870–7. doi: 10.1021/acs.analchem.1c02420 (PMC8655737; doi:10.1021/acs.analchem.1c02420)
Supplement: Supplementary file 1 — ac1c02420_si_001.pdf [file ac1c02420_si_001.pdf]

Supporting Information

When Red Turns Black: Influence of the 79 AD Volcanic Eruption and Burial Environment on the Blackening/Darkening of Pompeian Cinnabar

Silvia Pérez-Diez,[*,†] Africa Pitarch Martí,[‡,§] Anastasia Giakoumaki,[†,‖] Nagore Prieto-Taboada,[†] Silvia Fdez-Ortiz de Vallejuelo,[†] Alberta Martellone,[⁑] Bruno De Nigris,[⁑] Massimo Osanna,[○,ᵡ] Juan Manuel Madariaga[†,⊥] and Maite Maguregui[*,¥]

[†]Department of Analytical Chemistry, Faculty of Science and Technology, University of the Basque Country UPV/EHU, P.O. Box 644, 48080 Bilbao, Basque Country, Spain, [*]silvia.perezd@ehu.eus

[‡]Departament d'Arts i Conservació-Restauració, Facultat de Belles Arts, Universitat de Barcelona, Pau Gargallo, 4, 08028 Barcelona, Catalonia, Spain

[§]IAUB. Institut d'Arqueologia UB. Facultat de Geografia i Història. UB C/Montalegre 6-8 08001 Barcelona, Catalonia, Spain.

[‖]Institute of Electronic Structure and Laser – Foundation for Research and Technology, Nikolaou Plastira 100, Vassilika Vouton, 70013 Heraklion, Crete, Greece

[⁑] Applied Research Laboratory of the Archaeological Park of Pompeii, via Plinio 4, 80045 Pompeii, (NA), Italy

[○]Former General Director of the Archaeological Park of Pompeii, via Plinio 4, 80045 Pompeii, (NA), Italy

[ᵡ]Director-General of the Directorate-General of Museums, via di San Michele 22, 00153 Rome, Italy

[⊥]Unesco Chair on Cultural Landscape and Heritage, University of the Basque Country UPV/EHU, P.O. Box 450, 01008 Vitoria-Gasteiz, Basque Country, Spain

[¥]Department of Analytical Chemistry, Faculty of Pharmacy, University of the Basque Country UPV/EHU, P.O. Box 450, 01008 Vitoria-Gasteiz, Basque Country, Spain, [*]maite.maguregui@ehu.eus

*Corresponding authors: silvia.perezd@ehu.eus, maite.maguregui@ehu.eus

Keywords: cinnabar, Pompeii, Raman spectroscopy, calomel, manganese oxide

Table of Content

Supplementary figures

Figure S1 to S9

Supplementary tables

Table S1 to S4

INSTRUMENTATION

In-situ measurements and instrumentation

Portable Raman spectroscopy

The portable innoRam™ Raman spectrometer ($B_{\&}WTEK_{INC.}$, Newark, USA) implements a controller of the laser power (a scale from 0 to 100 % of the total power of the laser). It also includes a two-dimensional charge coupled device (CCD) to detect the dispersed Raman signal, which is Thermoelectric Cooled (TC) to −20 °C to maximize the dynamic range by reducing dark current. A back-thinned CCD is used to obtain 90 % quantum efficiency via collection of incoming photons at wavelengths that would not pass through a front illuminated CCD. With this instrument, the spectra were acquired between 65 and 3000 cm^{-1} at 4.0 cm^{-1} spectral resolution (measured at 912 nm). The spectral acquisition and data treatment was performed using the BWSpec™ v.4.0215 ($B_{\&}WTEK_{INC.}$, Newark, USA) and OMNIC 7.2 (Thermo, Massachusetts, USA) software.

To mount the Raman probe in the motorized tripod (MICROBEAM S.A. Barcelona, Spain), it was inserted in a portable video-microscope to perform the micro-focusing on the pigment layer. For stability reasons, measurements were acquired under a 20× (8.8-mm working distance and 105-µm laser beam spot size) objective lens when in-situ measurements were conducted outdoors (Archaeological Park of Pompeii) and under a 50× (3.68-mm working distance and 42-µm laser beam spot size) objective lens when they were conducted indoors (MANN).

Handheld Energy Dispersive X-ray Fluorescence spectrometry (HH-EDXRF)

The size of the emitted X-ray beam of the XMET5100 (Oxford Instruments, UK) HH-EDXRF spectrometer is 9 mm. The analyzer includes a high-resolution silicon drift detector (SDD), able to give a resolution of 150 eV (FWHM of the Mn K_{α} line). Besides, the analyzer contains a PDA to control the spectrometer and save the spectral and quantitative information. Spectral information of heavier elements (Z>21) was acquired working with the tube at 45 kV and 15 µA. To improve the limit of detection of the light elements (especially Cl), additional spectra were acquired at lower voltage (13 kV) and higher current (45 µA). All the measurements were acquired during 100 s. After a proper deconvolution of the spectra, and in order to mainly overcome the interference of Rh L_{α} line against the Cl K_{α} line, the counts of each element were obtained using the ArTax 8.476 software (Bruker Nano GmbH, Berlin, Germany). To perform a comparative study of the S and Cl information obtained in the different walls or panels under study net areas of Cl and S (K_{α} lines) were normalized against the portion of the background scatter close to these two K_{α} lines of each element. For comparison purpose net areas of the elements were also normalized to the total counts of the spectra. As very similar or coincident results and comparative tendencies were obtained, in this work, the normalized counts obtained using the portion of the background scatter close to Cl and S K_{α} line is shown. The normalization procedure will minimize the geometrical effects derived from irregularities from the surface of the paintings, being possible to compare the normalized net areas of the fluorescence peaks of adjacent painting areas.

Benchtop instrumentation

Raman microscopy

The inVia confocal Raman microscope (Renishaw, Gloucestershire, UK) is equipped with a Peltier-cooled CCD detector (-70 °C). The spectrometer is coupled to DMLM Leica microscope, which can use a great variety of long-range lens lens (5×, 20×, 50× and 100×). The confocality allows obtaining the maximum lateral resolution of the microscope. The microscope is equipped with a motorized XYZ positioning stage with integrated position sensors on the X and Y axes (Renishaw).

To prevent from possible thermal decomposition of the area under study, spectra were acquired from the lower laser power (around 0.4 mW) increasing it gradually (no more than 20 mW) until the improvement of the spectrum was achieved. Before and after the measurement, the integrity of the area under study and the repeatability of the spectral result while the laser power was increased were checked.

Micro EDXRF spectrometry (µ-EDXRF)

The M4 TORNADO (Bruker Nano GmbH, Berlin, Germany) µ-EDXRF spectrometer implements two XFlash silicon drift detectors achieving an energy resolution of 145 eV for Mn-K_{α} line. The poly-capillary focusing optics offers a variation of the spot size as a function of the energy being 17 µm at 2.3 keV and 32 µm at 18.3 keV.

The focusing process was conducted using two video microscopes, the first explores the sample under a low magnification (1 cm^2 area), while the second performs the final focusing (1 mm^2 area) to conduct the analysis.

The XRF elemental images (hypermaps) were acquired at 20 ms, 2 scans and a step size of 20 µm. To construct the elemental images a previous deconvolution of the signals in the sum spectrum representing the whole mapped area was conducted. After that, the distribution map of each element was represented as a function of the intensity of each K_α line.

Table S1. List of cinnabar-based samples and paintings from Pompeii with information about their provenance, location, orientation, style, type of exposure and analyses conducted on them.

Reference	Type of sample/painting				House	Location	Wall	Style	Type of exposure			Analysis				
	Raw fragment	Cross-section	Panel painting[a]	Mural painting[b]					79 AD volcanic eruption	Modern atmosphere	Not exposed since their deposition in the pit[c]	RS	SEM-EDS	XPS and TOF-SIMS	µ-ED XRF	HH-ED XRF
ATT 2007/14	x	x			HML	*triclinium*	North	4th	x	x		x	x			
16/56	x	x			HML	*triclinium*	South	4th	x	x		x	x			
3T	x	x			HML	waste pit in the *viridarium*	Undet.	2nd			x	x		x		
Red A	x				HML	waste pit in the *viridarium*	Undet.	2nd			x	x			x	
9206			x		HML	*triclinium*	South	4th	x							x
9285			x		HML	*triclinium*	South	4th	x							x
8992			x		HML	*triclinium*	East	4th	x							x
9103			x		HML	summer *triclinium*	West	4th	x			x				
Triclinium				x	HML	*triclinium*	East and south	4th	x	x		x				x
6		x			HA	*oecus*	-	4th	x	x		x				
17		x			HA	*ala*	South	4th	x	x		x				

18		x			HA	*ala*	South	4th	x	x		x	x			
Ala and *oecus*				x	HA	*ala* and *oecus*	Undet.	4th	x	x		x				
Exedra				x	HGC	*exedra*	South	3rd	x	x		x				x

a: painting stored at the MANN; b: painting analyzed at its original location; c: time unknown; HML: House of Marcus Lucretius, HA: House of Ariadne, HGC: House of the Golden Cupids; RS: Raman spectroscopy; SEM-EDS: Scanning electron microscopy coupled with an energy dispersive spectrometer; XPS: X-ray Photoelectron Spectroscopy; TOF-SIMS: Time-of-Flight Secondary Ion Mass Spectrometry; μ-EDXRF: micro energy dispersive X-ray fluorescence spectrometry; HH-EDXRF: handheld energy dispersive X-ray fluorescence.

Table S2. Summary of the compounds identified by in-situ and laboratory-based Raman spectroscopy on cinnabar paintings from Pompeii.

Reference	Type of analysis		Identified Compounds													Interpretation
	In-situ	Lab	Anatase	Calcite	Calomel	Cinnabar	Dolomite	Egyptian Blue	Gypsum	Goethite	Hematite	Manganese oxide	Magnetite	Tridimite	Wax	
ATT2007/14		x	x	x	x	x			x		x				x	Blackening
16/56		x		x	x	x			x		x					Blackening
3T		x	x	x		x		x		x	x					Darkening
Red A		x		x		x						x				Darkening
Panel 9103	x			x		x			x							Blackening
Triclinium HML	x			x	x	x			x	x	x		x			Blackening
6		x		x	x	x				x				x		Blackening
17		x		x	x				x		x					Blackening
18		x		x		x	x		x		x					Blackening
Ala and *oecus* HA	x				x	x			x							Blackening

Exedra<br><br>HGC	x			x	x	x			x	x					x	Blackening

Table S3. Raman bands detected in sample ATT2007/14 ascribable to a saturated wax and proposed vibrational assignment for each band.

Raman band / cm^{-1}	Proposed vibrational assignment [1]
142	τ(COH)
890	$\rho(CH_2)$, *d*(COC)
920	ρ(CH3)
1063	n(C-O)
1174	n(CC)
1296	*d*(CH_2) twisting
1372	*d*(CH_2)
1419	*d*(CH_2)
1441	*d*(CH_2) scissoring
1463	*d*(CH_2) scissoring
1734	n(C=O)
2723	n$(CH_3$-$CH_2)$
2849	n(CH_2) symmetrical
2884	n(CH_2) asymmetrical

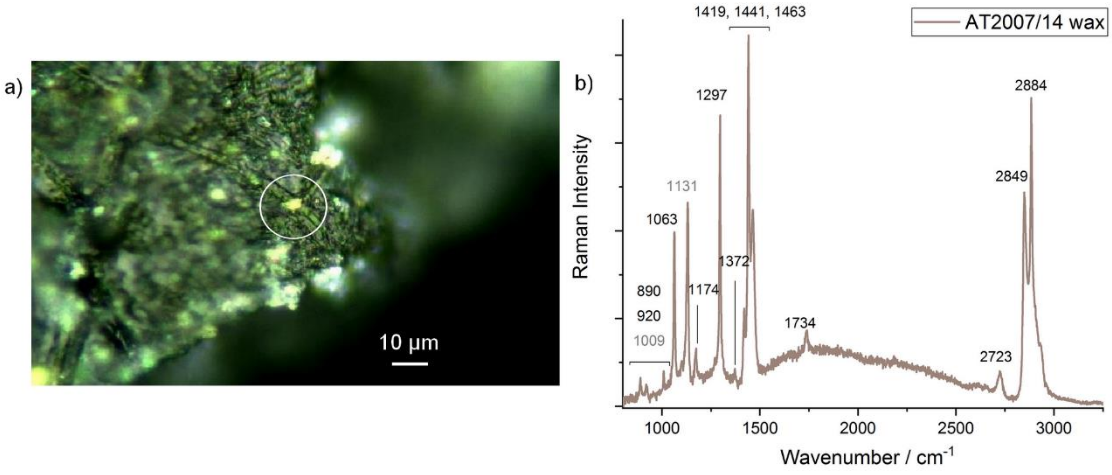

Figure S1. a) Optical micrograph of sample ATT2007/14 (*triclinium*, Room 16, the House of Marcus Lucretius). b) Selected Raman spectrum showing the characteristic Raman bands of a saturated wax (890, 920, 1063, 1174, 1297, 1372, 1419, 1441, 1463, 1734, 2723, 2849, 2884 cm^{-1}) and gypsum (1009 and 1131 cm^{-1}),

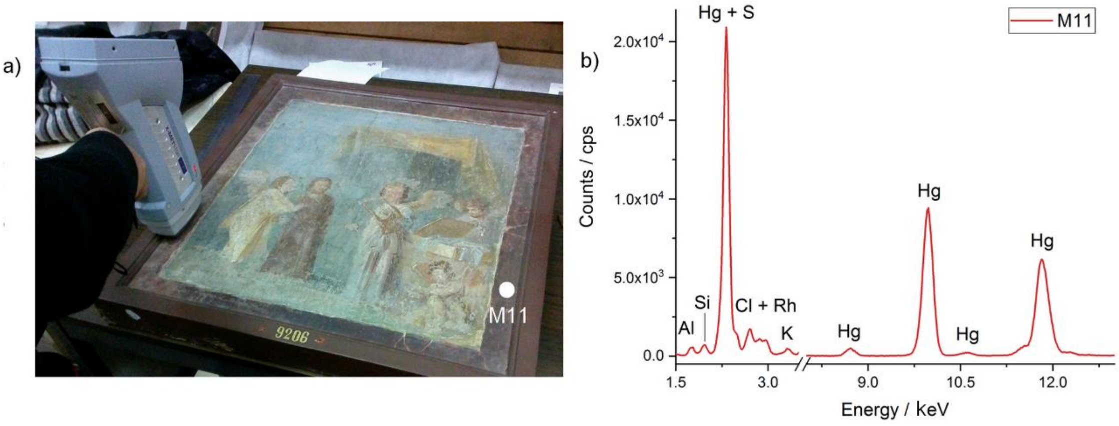

Figure S2. a) Close-up view of the analytical set-up for the in-situ HH-EDXRF measurements and location of M11 within panel 9206. b) Selected EDXRF spectrum of the blackened red cinnabar frame of the panel (spot M11), showing the presence of Hg, S and Cl.

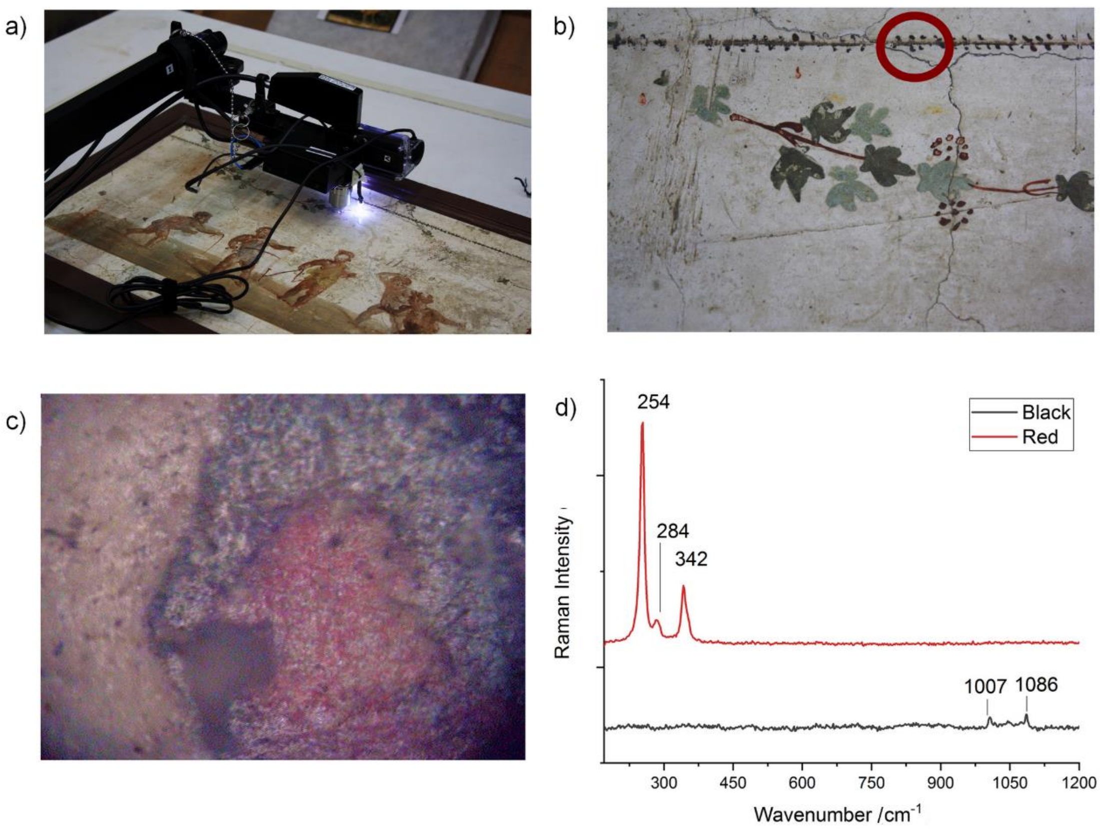

Figure S3. a) Close-up view of the analytical set-up for the in-situ Raman measurements. b) Location of the blackened red cinnabar areas within the panel. c) Microscopic image showing the coexistence of red and blackened red areas. d) Selected Raman spectra of the blackened and intact red area on the garland of panel 9103 (MANN), showing the Raman bands of gypsum (1007 cm^{-1}) and calcite (1086 cm^{-1}) in the blackened cinnabar areas, and cinnabar (254, 284, 342 cm^{-1}) in the red ones.

Table S4. Normalized S and Cl counts obtained by HH-EDXRF in the measured areas on the south and east walls of the *triclinium* of the House of Marcus Lucretius.

Wall's orientation	Panel	Analyzed area	Normalized S (cps)	Normalized Cl (cps)
South	9191	Left side	4.1 ± 0.3	0.5 ± 0.2
	9191	Right side	4.0 ± 0.5	3.7 ± 0.3
	9285	Left side	2.0 ± 0.5	1.1 ± 0.8
	9285	Left area of the panel	16 ± 3	0.3 ± 0.2
	9285	Right area of the panel	30 ± 8	0.1 ± 0.1
	9285	Right side	13 ± 4	1.2 ± 0.3
	9206	Left side	19 ± 4	0.9 ± 0.3
	9206	Panel	29 ± 4	0.3 ± 0.1
	9206	Right side	9 ± 2	0.5 ± 0.2
East	8992	Left side	2.6 ± 0.6	0.2 ± 0.1
	8992	Left area of the panel	6 ± 3	0.2 ± 0.2
	8992	Right area of the panel	19 ± 10	0.3 ± 0.3
	8992	Right side	3 ± 1	0.6 ± 0.2

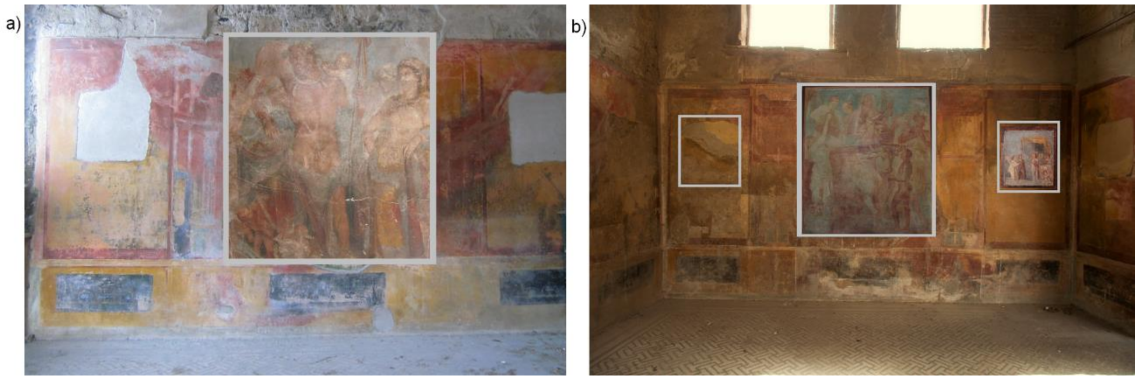

Figure S4. a) View of the eastern wall of the *triclinium* of the House of Marcus Lucretius, where the voids left by three figure panels, nowadays stored at MANN, are visible. Note the original position of panel 8992 on the center of the wall. b) View of the southern wall of the *triclinium* of the House of Marcus Lucretius, where the voids left by three figure panels (from left to right: 9191, 9285, 9206), nowadays stored at MANN, are visible. Note the original position of panel 9285 on the center and panel 9206 on the right side of the wall.

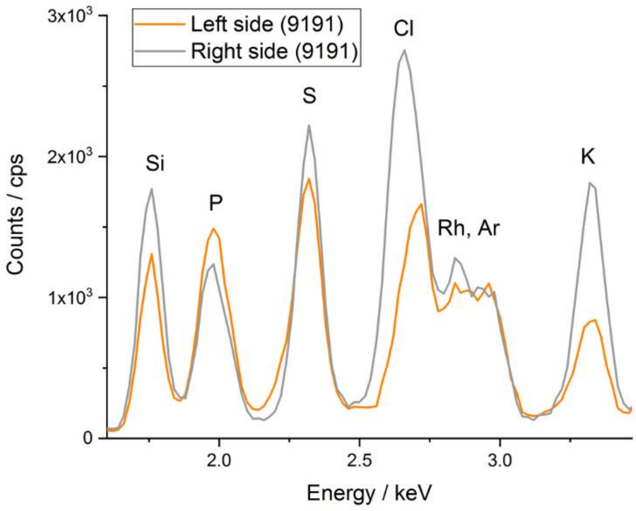

Figure S5. Selected EDXRF spectra of left and the right side of the void left by panel 9191 on the south wall of the *triclinium* (see Figure S4b). Note the higher intensity of the Cl peak in the spectrum of the right side, also noticeable in the normalized Cl counts reported in Table S4.

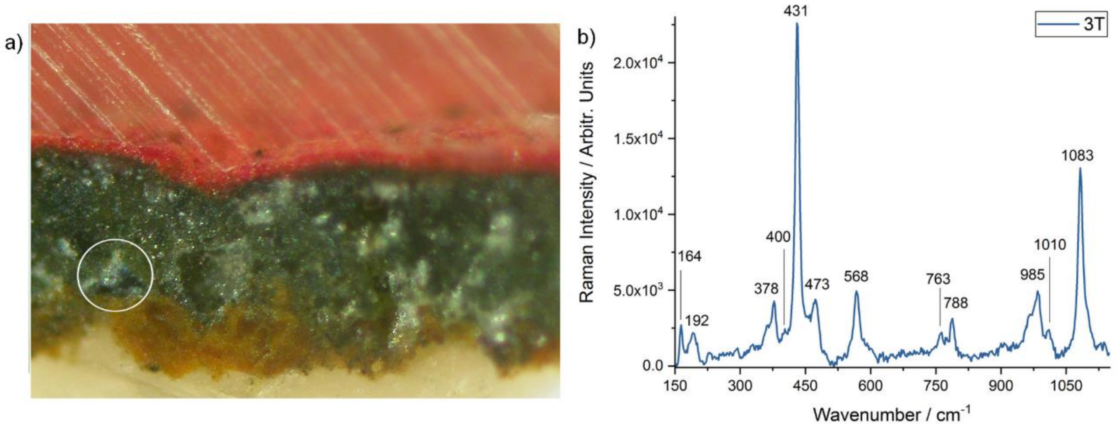

Figure S6. a) 100x image of the red painting layer (cinnabar) over a green layer composed of an Egyptian blue and goethite (300 and 386 cm^{-1}, spectrum not shown). b) Selected Raman spectrum of a blue grain in the sample, showing the characteristic Raman bands of Egyptian blue (112, 137, 164, 192, 378, 400, 431, 473, 568, 763, 788, 985, 1010 and 1083 cm^{-1}).

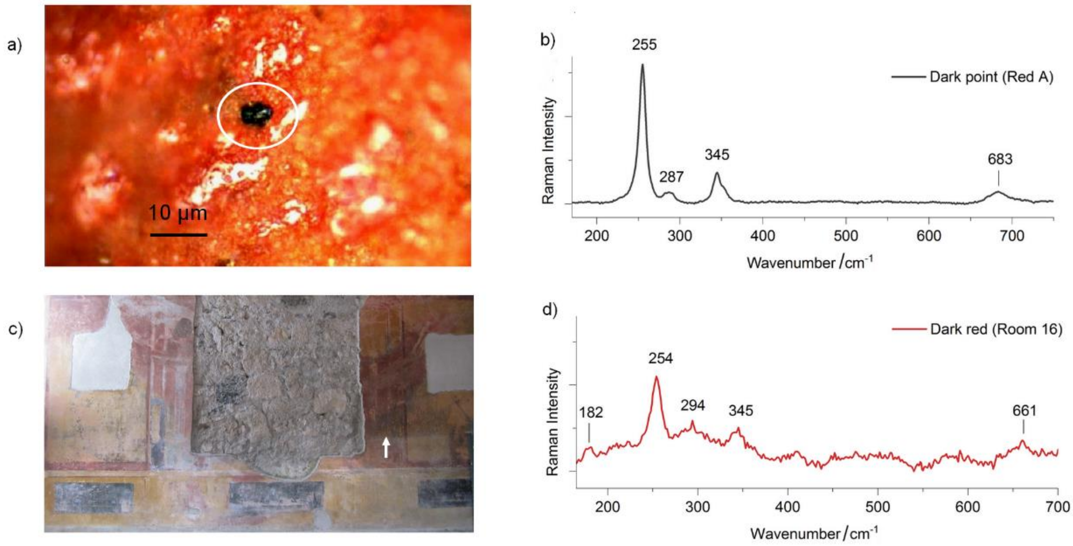

Figure S7. a) Optical micrograph of the dark spot on sample Red A. b) Selected Raman spectrum of a dark spot on the red layer of sample Red A showing the Raman bands of cinnabar (255, 287, 345 cm^{-1}) and an additional broad band at 683 cm^{-1}. c) View of the eastern wall of the *triclinium*, where both the dehydration of yellow into red ocher and the darkening of the red pigments are visible. d) Raman spectrum of a darkened red area of the eastern wall of the *triclinium* showing the Raman bands of cinnabar (254, 294, 345 cm^{-1}) and other bands that can be ascribed to MnO_2 (182, 661 cm^{-1})[2,3] or magnetite (661 cm^{-1})[4].

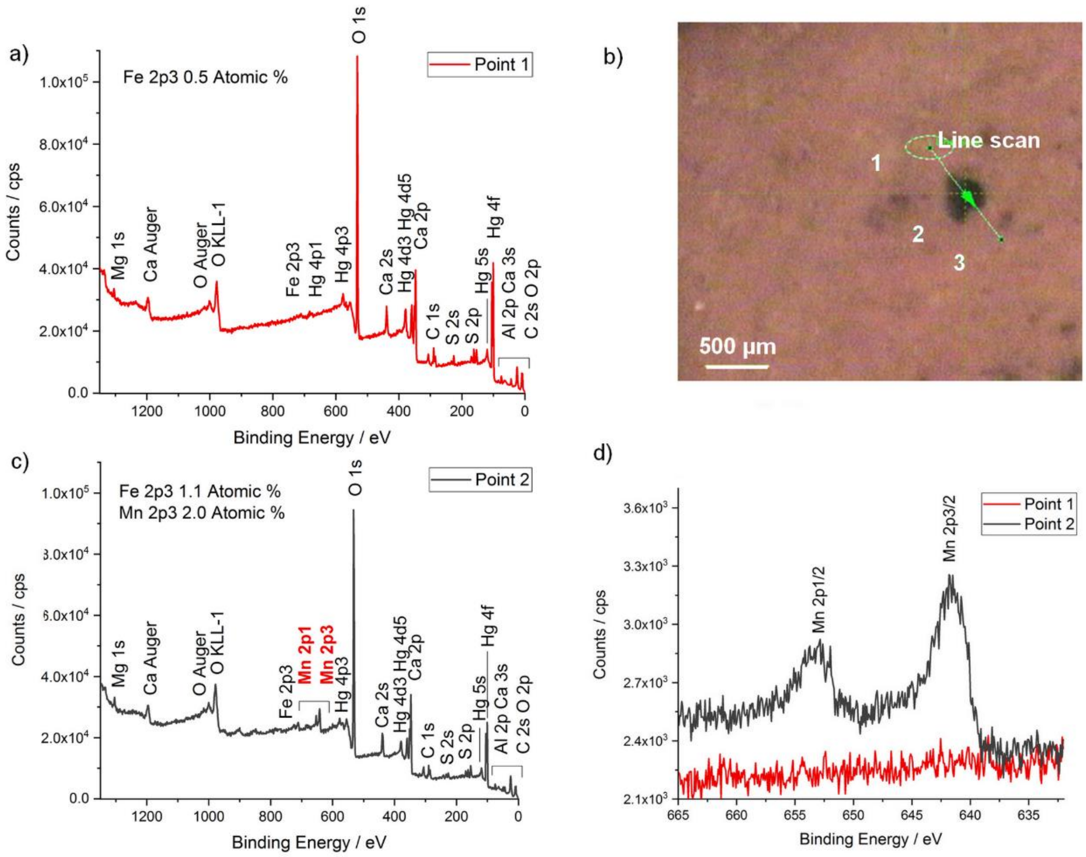

Figure S8. a) XPS spectrum acquired in the red background of sample 3T, corresponding to point 1. b) View of the surface of sample 3T, where points 1 and 3 were measured on the red cinnabar background and point 2 on the dark spots. c) XPS spectrum acquired in the dark spot, corresponding to point 2. d) High resolution XPS spectrum (Mn 2p1 and 2p3) acquired on the dark spot (point 2).

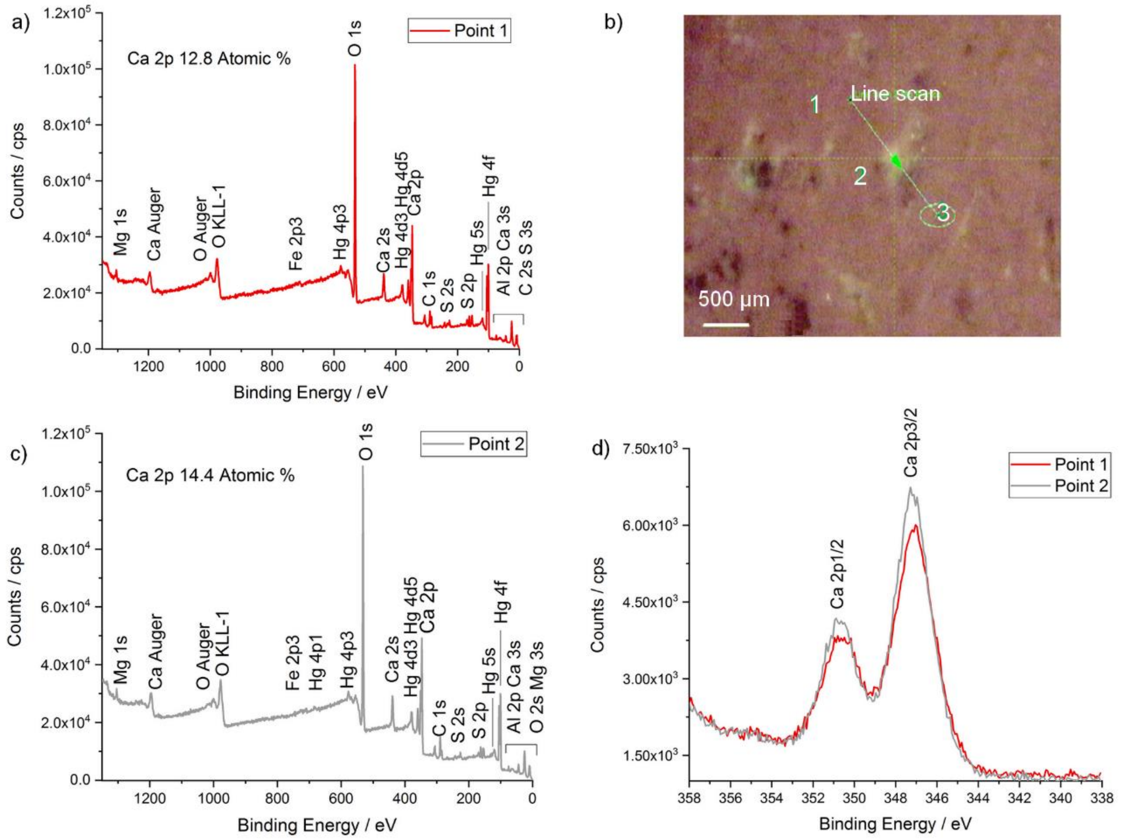

Figure S9. a) XPS spectrum acquired in the red background of sample 3T, corresponding to point 1. b) View of the surface of sample 3T, where points 1 and 3 were measured on the red cinnabar background and point 2 on the whitish area. c) XPS spectrum acquired in the whitish area, corresponding to point 2. d) High resolution XPS spectrum (Ca 2p) acquired on the whitish area (point 2).

REFERENCES

(1) Edwards, H. G. M.; Falk, M. J. P. Fourier-Transform Raman Spectroscopic Study of Unsaturated and Saturated Waxes. *Spectrochim. Acta. A. Mol. Biomol. Spectrosc.* **1997**, *53* (14), 2685–2694. https://doi.org/10.1016/S1386-1425(97)00161-3 .

(2) Sepúlveda, M.; Gutiérrez, S.; Vallette, M. C.; Standen, V. G.; Arriaza, B. T.; Cárcamo-Vega, J. J. Micro-Raman Spectral Identification of Manganese Oxides Black Pigments in an Archaeological Context in Northern Chile. *Herit. Sci. Lond.* **2015**, *3* (1), 1–6. http://dx.doi.org.ehu.idm.oclc.org/10.1186/s40494-015-0061-2.

(3) Gao, T.; Glerup, M.; Krumeich, F.; Nesper, R.; Fjellvåg, H.; Norby, P. Microstructures and Spectroscopic Properties of Cryptomelane-Type Manganese Dioxide Nanofibers. *J. Phys. Chem. C* **2008**, *112* (34), 13134–13140. https://doi.org/10.1021/jp804924f.

(4) Faria, D. L. A. de; Silva, S. V.; Oliveira, M. T. de. Raman microspectroscopy of some iron oxides and oxyhydroxides. *J. Raman Spectrosc.* **1997**, *28* (11), 873–878. https://doi.org/10.1002/(SICI)1097-4555(199711)28:11<873::AID-JRS177>3.0.CO;2-B.
